# Supplementary figures and images for: Integrated transcriptomic and proteomic analysis of pathogenic mycobacteria and their esx-1 mutants reveal secretion-dependent regulation of ESX-1 substrates and WhiB6 as a transcriptional regulator
Source: PLoS One. 2019 Jan 23;14(1):e0211003. doi: 10.1371/journal.pone.0211003 (PMC6343904; doi:10.1371/journal.pone.0211003)

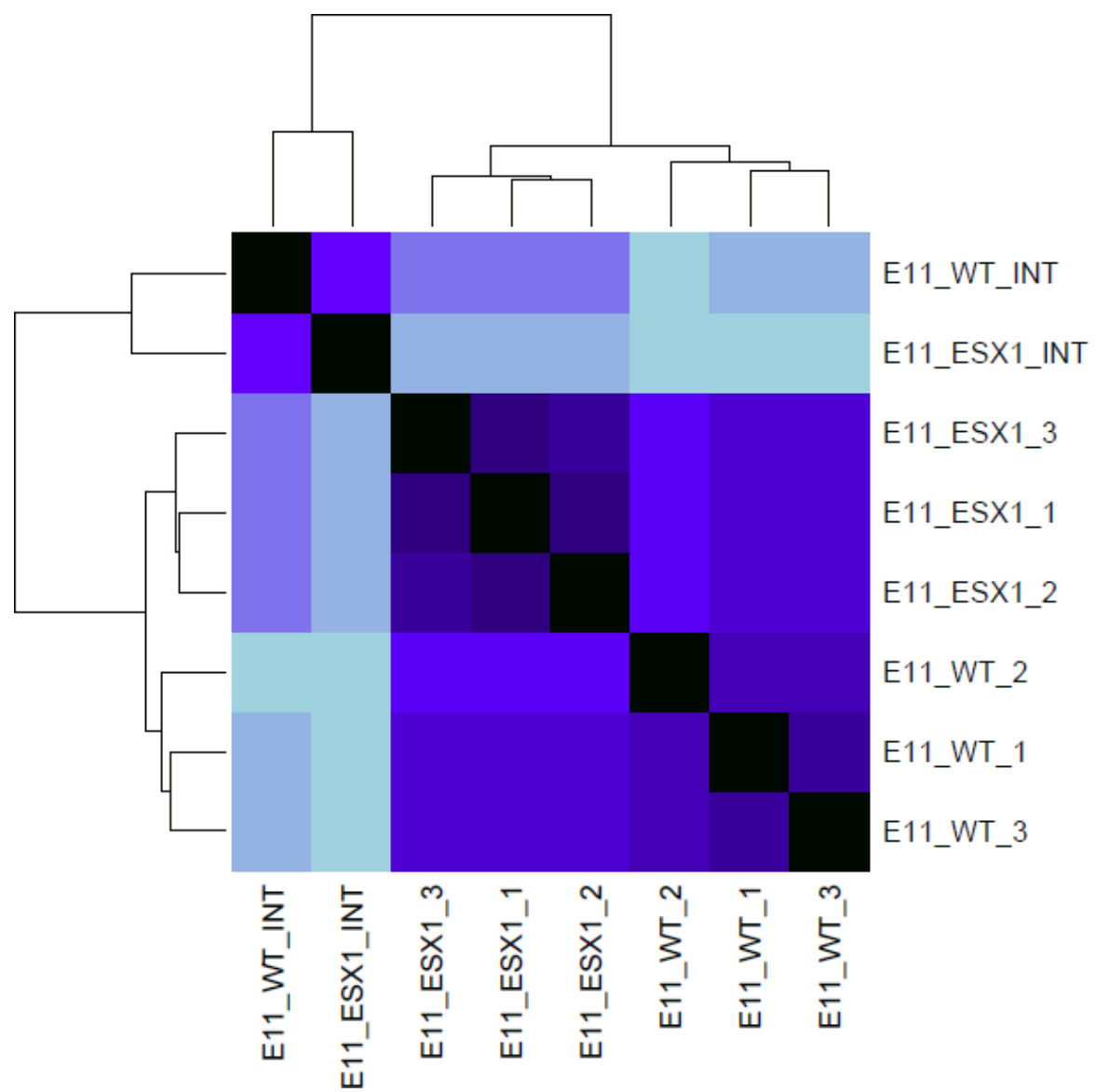

S1 Fig

Supplement: S1 Fig — (PDF) [file pone.0211003.s009.pdf]

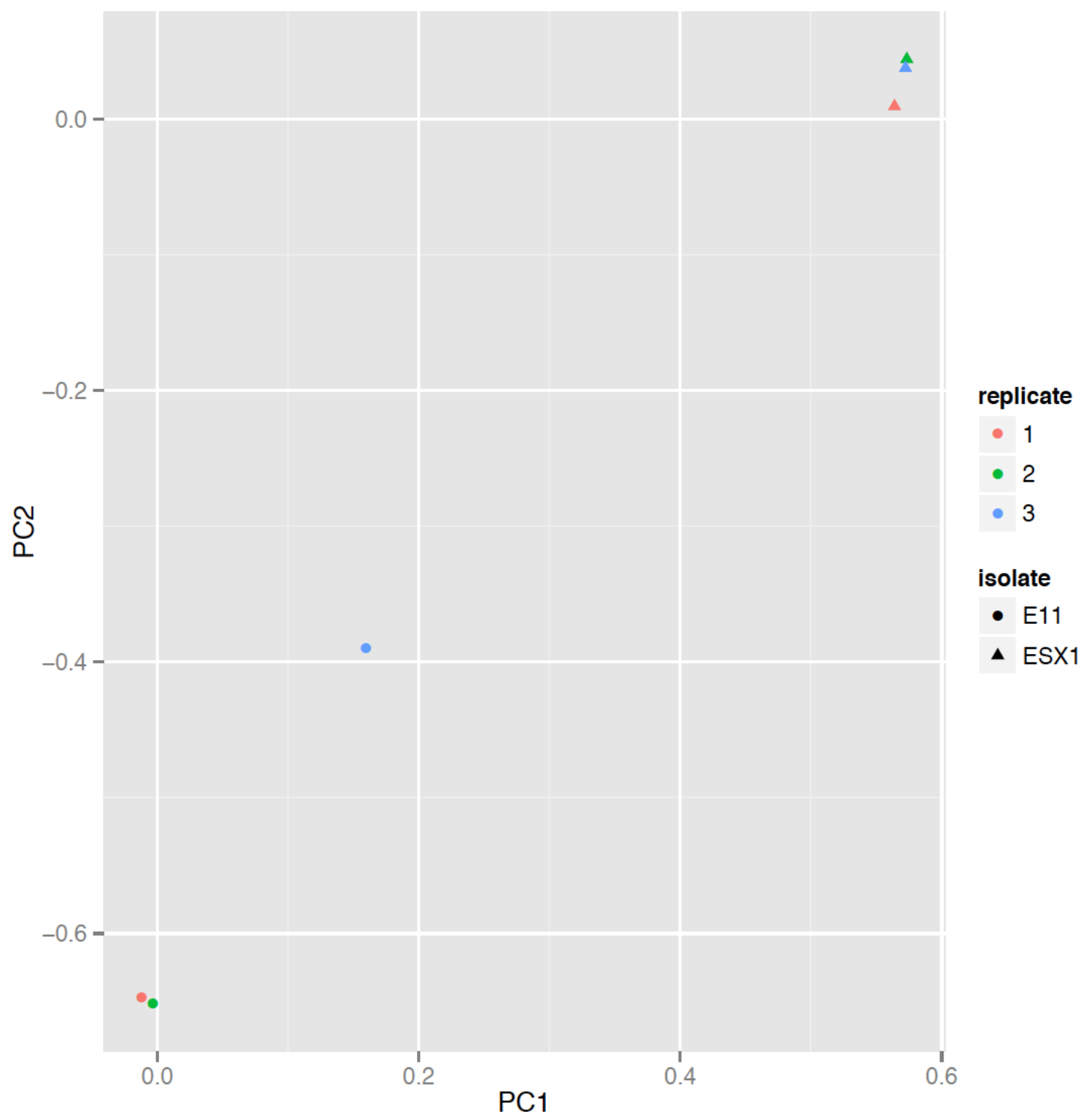

S2 Fig

Supplement: S2 Fig — PCA mapping showed clustering of biological replicates of the E11 wild-type and eccCb1 mutant strains. (PDF) [file pone.0211003.s010.pdf]

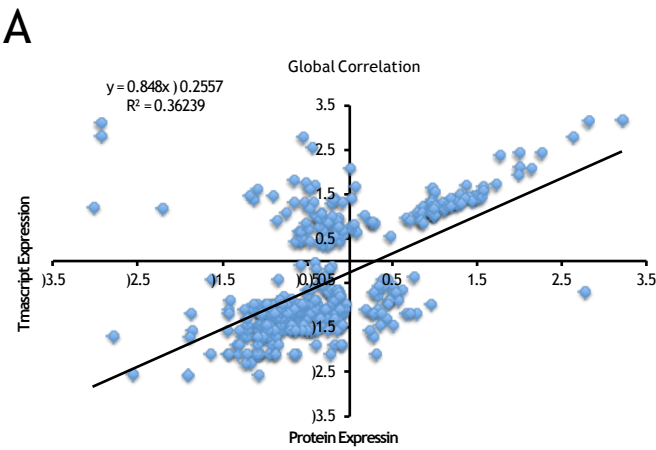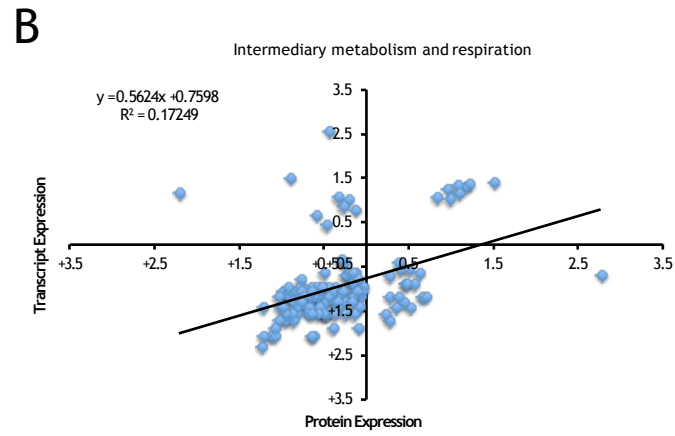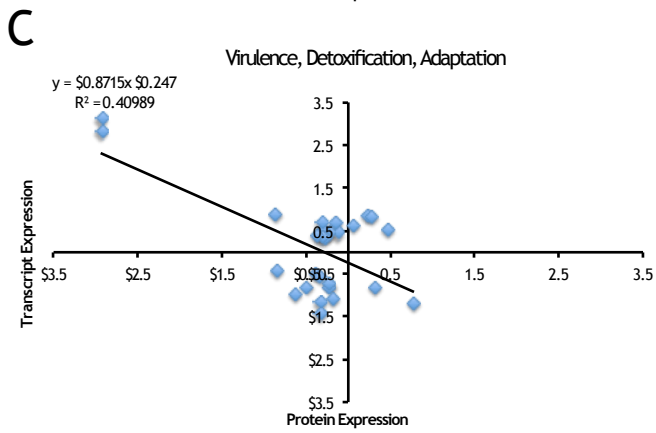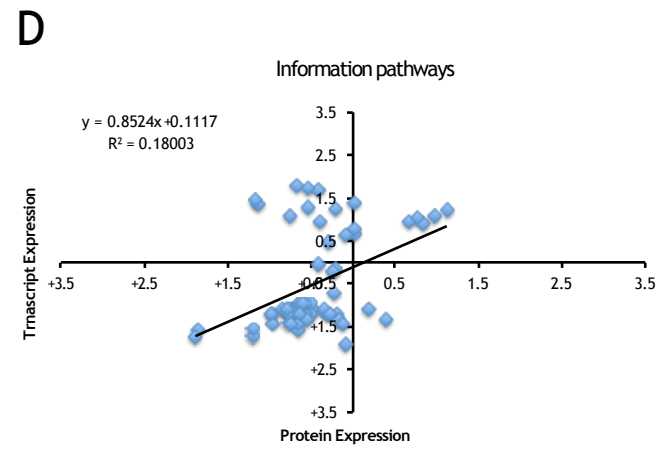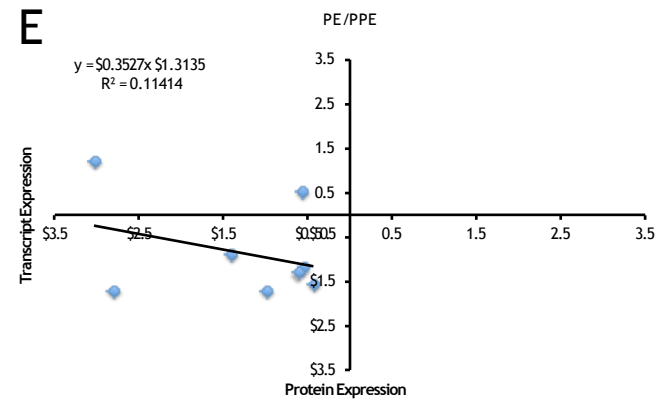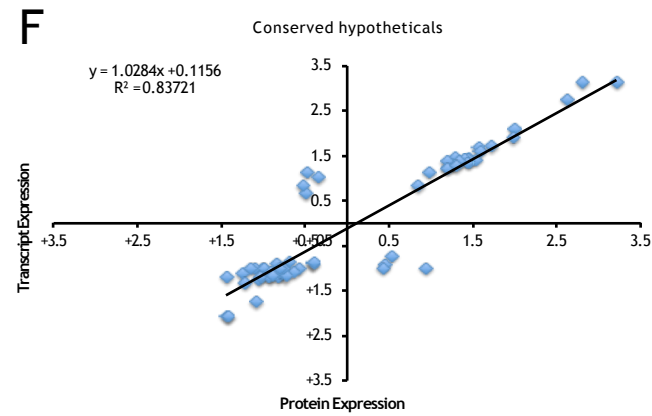

Supplement: S3 Fig — (A) Scatterplot of the relationship between differentially expressed genes quantified in both data sets. (B-F) Scatterplots for protein and gene transcript expression classified by functional categories. Scatterplots display the rectilinear equation and the Pearson correlation coefficient (R2). (PDF) [file pone.0211003.s011.pdf]

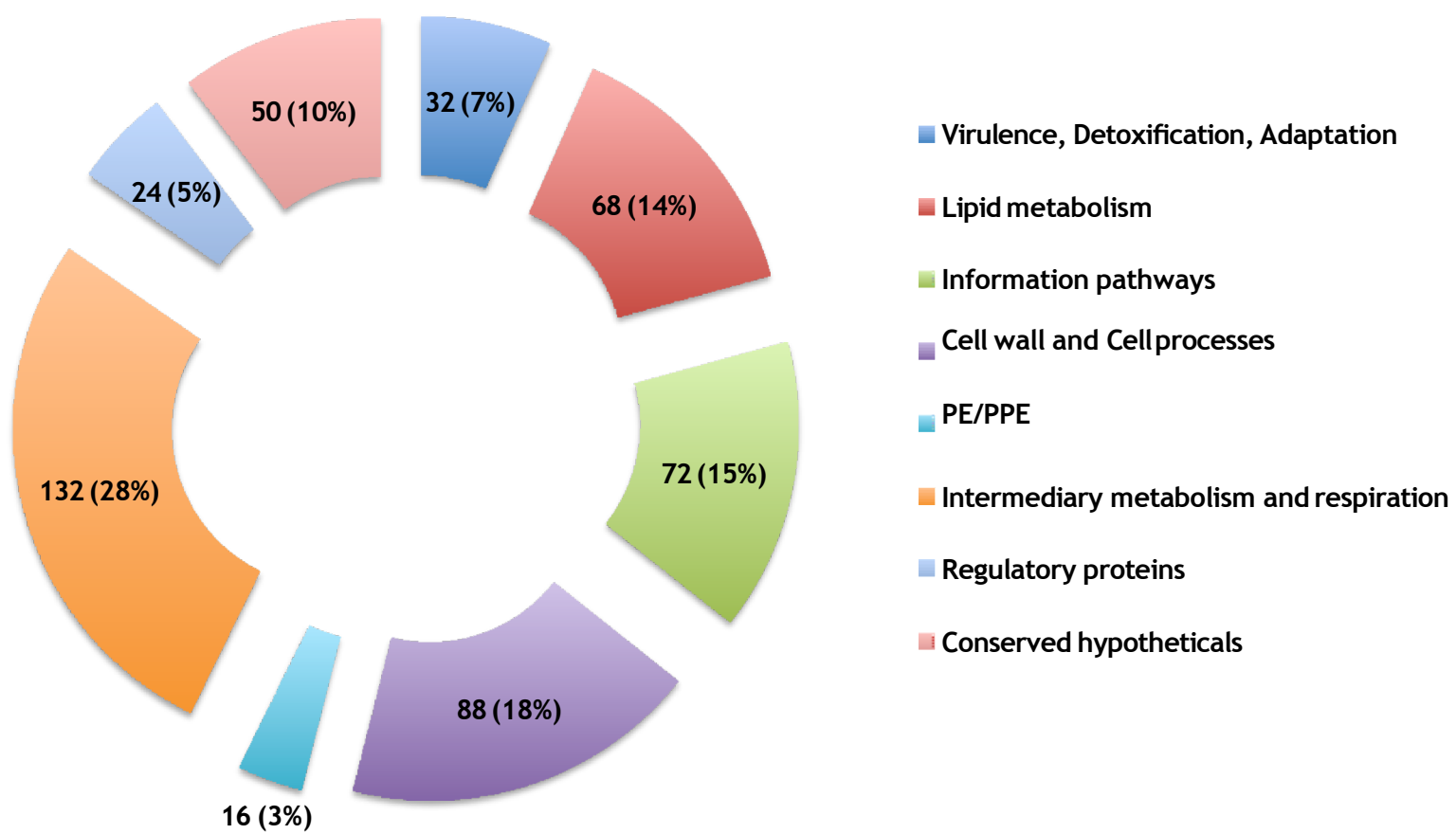

S4 Fig

Supplement: S4 Fig — Genes exhibiting differential expression at the RNA and protein levels were grouped according to the MarinoList classification (http://mycobrowser.epfl.ch/marinolist.html). (PDF) [file pone.0211003.s012.pdf]

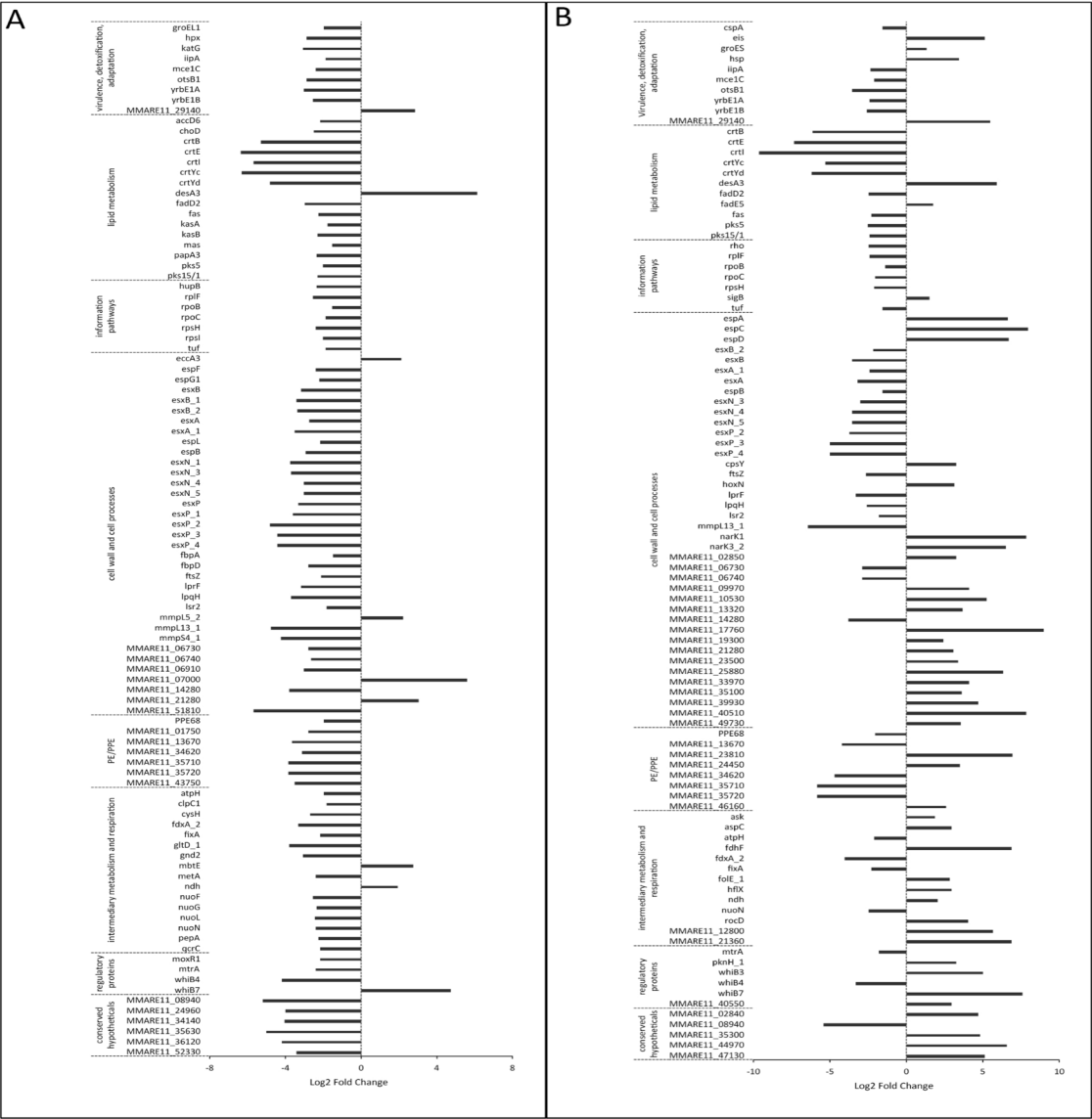

S5 Fig

Supplement: S5 Fig — Within each group, genes are ranked in ascending order by P-value. (A). Top 100 annotated genes from the M. marinum E11 strain that were the most differentially expressed in the M. marinum wild-type strain E11 during infection of primary macrophages. Bar chart of log2-fold changes for individual genes (tags, left). (B). Top 100 annotated genes from the M. marinum E11 strain that were the most differentially expressed in the M. marinum eccCb1 transposon mutant compared to the isogenic wild-type strain E11 during infection of primary macrophages (tags, left). Bar chart of log2-fold changes for individual genes. (PDF) [file pone.0211003.s013.pdf]

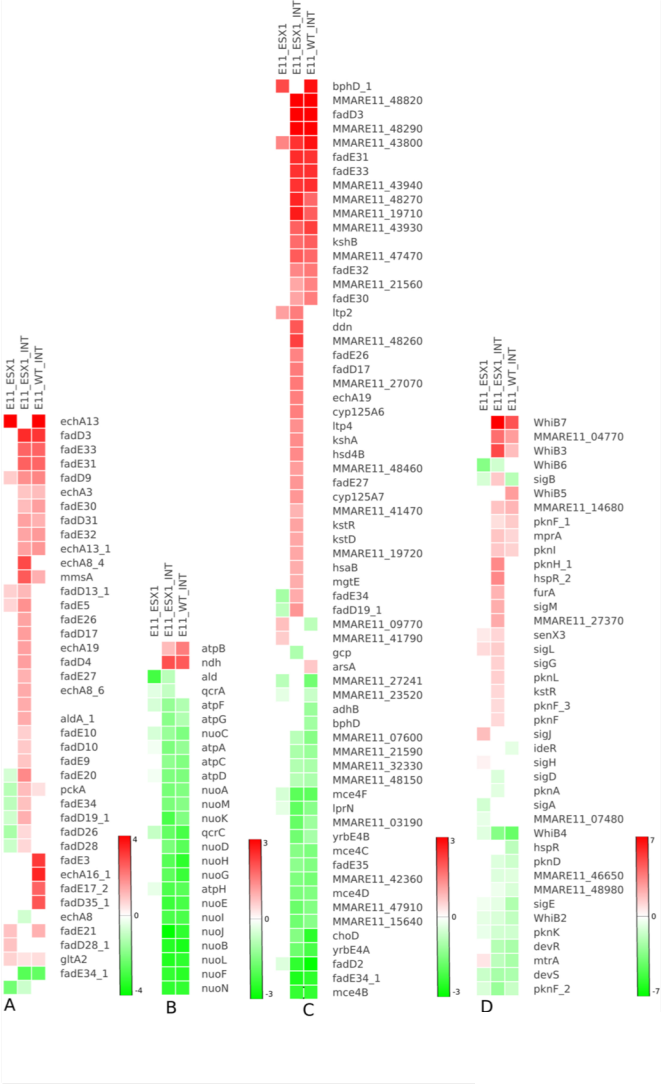

S6 Fig

Supplement: S6 Fig — (A) Catabolism of fatty acids. Genes were selected based on their annotation and ordered based on expression. (B) Energy generation and NAD+ regeneration. Genes were selected based on their annotation and ordered based on expression. (C) Genes of the kstR regulon, which are required for uptake and metabolism of cholesterol [61, 77]. (D) Transcriptional regulation. Genes were selected based on their annotation and ordered based on expression. (PDF) [file pone.0211003.s014.pdf]

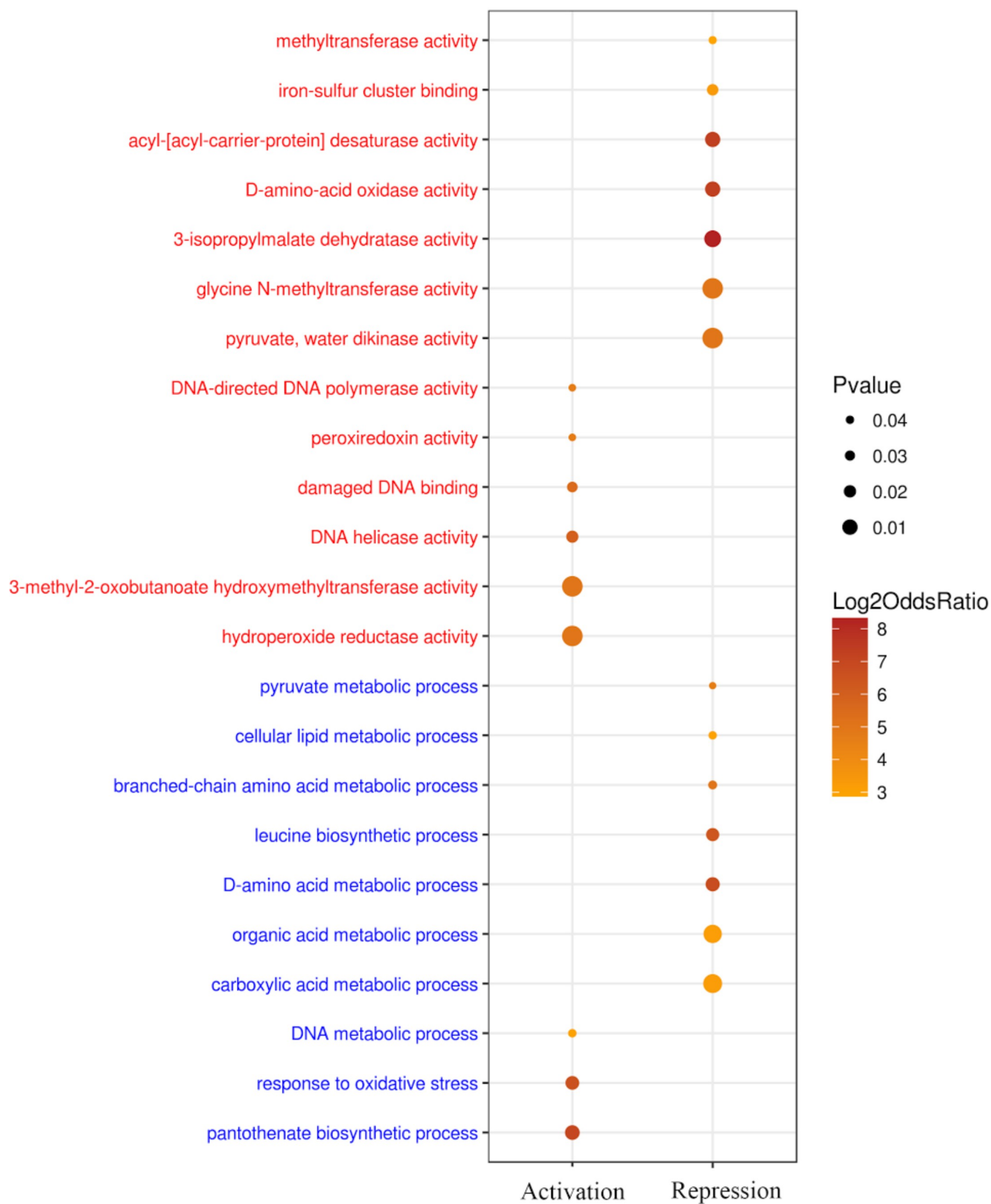

S7 Fig

Supplement: S7 Fig — The molecular function GO terms are in red, while the biological process terms are in blue. (PDF) [file pone.0211003.s015.pdf]
